# Therapeutic Interventions for Countering Leishmaniasis and Chagas’s Disease: From Traditional Sources to Nanotechnological Systems

**DOI:** 10.3390/pathogens8030119

**Published:** 2019-08-01

**Authors:** Eliana B. Souto, João Dias-Ferreira, Sara A. Craveiro, Patrícia Severino, Elena Sanchez-Lopez, Maria L. Garcia, Amélia M. Silva, Selma B. Souto, Sheefali Mahant

**Affiliations:** 1 Department of Pharmaceutical Technology, Faculty of Pharmacy, University of Coimbra (FFUC), Pólo das Ciências da Saúde, Azinhaga de Santa Comba, 3000-548 Coimbra, Portugal; 2 CEB – Centre of Biological Engineering, University of Minho, Campus de Gualtar, 4710-057 Braga, Portugal; 3 Faculty of Health Sciences, University Fernando Pessoa, Rua Carlos da Maia, 296, Paranhos, 4200-150 Porto, Portugal; 4 Laboratory of Nanotechnology and Nanomedicine (LNMED), Institute of Technology and Research (ITP), Av. Murilo Dantas, 300, Aracaju 49010-390, Brazil; 5 University of Tiradentes (UNIT), Industrial Biotechnology Program, Av. Murilo Dantas 300, Aracaju 49032-490, Brazil; 6 Department of Pharmacy, Pharmaceutical Technology and Physical Chemistry, Faculty of Pharmacy, University of Barcelona, 08028 Barcelona, Spain; 7 Institute of Nanoscience and Nanotechnology (IN2UB), University of Barcelona, 08028 Barcelona, Spain; 8 Centro de Investigación Biomédica en Red de Enfermedades Neurodegenerativas (CIBERNED), University of Barcelona, 08028 Barcelona, Spain; 9 Departamento de Biologia e Ambiente, Universidade de Trás-os-Montes e Alto Douro (UTAD), P.O. Box 1013, 5001-801 Vila Real, Portugal; 10 Centro de Investigação e de Tecnologias Agro-Ambientais e Biológicas (CITAB-UTAD), 5001-801 Vila Real, Portugal; 11 Department of Endocrinology of Braga Hospital, Sete Fontes, 4710-243 São Victor, Braga, Portugal; 12 Department of Pharmaceutical Sciences, Maharshi Dayanand University, Rohtak, Haryana 124001, India

**Keywords:** Neglected diseases, drug delivery systems, antiprotozoal agents, Chagas’s disease, leishmaniasis

## Abstract

The incidence of neglected diseases in tropical countries, such as Leishmaniasis and Chagas’s disease, is attributed to a set of biological and ecological factors associated with the socioeconomic context of developing countries and with a significant burden to health care systems. Both Leishmaniasis and Chagas’s disease are caused by different protozoa and develop diverse symptoms, which depend on the specific species infecting man. Currently available drugs to treat these disorders have limited therapeutic outcomes, frequently due to microorganisms’ drug resistance. In recent years, significant efforts have been made towards the development of innovative drug delivery systems aiming to improve bioavailability and pharmacokinetic profiles of classical drug therapy. This paper discusses the key facts of Leishmaniasis and Chagas’s disease, the currently available pharmacological therapies and the new drug delivery systems for conventional drugs.

## 1. Introduction

In recent decades, the medical and pharmaceutical fields have been offering breakthrough solutions to drug delivery problems by nanotechnological innovations. However, the world still faces the demand for improved solutions to deal with spreadable pathologies, commonly referred to as neglected diseases (NDs). The latter are defined as a group of infections of bacterial, parasitic, protozoan, helminthic and viral origin, essentially affecting tropical countries, economically unfavorable, and where NDs are endemic. Most of these infections are caused by protozoa, for which transmission is carried out by means of vectors. Therapeutic solutions still rely on a small range of products, mostly chemically unstable or obsolete [1,2]. 

NDs are a result of biological and ecological circumstances, commonly associated with adverse socioeconomic conditions and with low development of affected countries worldwide [3]. 

Owing to the trends of the pharmaceutical industry in pursuing more profitable markets, these illnesses represent a great concern of health care systems [2,4]. The impact of NDs is huge as they are estimated to cause an average loss of 534,000 lives every year, leading to an economic cargo of billions of dollars annually. The overall occurrence of these pathologies is enhanced by the deprivation of authorized vaccines, thereby requiring the need for more reliable and infallible drugs to be used in their treatment. In addition, where the access to medication is possible, its effective use is becoming further restricted due to parasite resistance [5]. To overcome this limitation, efforts have been made to develop new therapeutic approaches [6]. Despite the number of currently ongoing governmental health programs to target these pathologies, the successful rate in their eradication is still below expectations. Table 1 lists the 13 most common tropical NDs [7,8,9]. 

Different regions worldwide are affected by different parasites, resulting in the need for more effective drugs. Still, the impact of economic investments to treat the affected populations along with the reduced expectation of economic return to the pharmaceutical industries are reasons for the limited research developments in this field. 

In the last 30 years, less than 1% of all new drugs were intended for the treatment of tropical diseases. As they are not therapeutic priorities, the capitals which could be targeted to the treatment of NDs have been applied to others of lower incidence, but with direct (economic) impact in, e.g., North America and Europe [10,11].

Given this scenario, some pharmaceutical companies have extended their portfolio on research and development of medicines into the sector of parasitic diseases, but not without previous identification of some goals, namely, reduced manufacturing price, high oral bioavailability, tolerability of adverse effects, possibility of co-administration with other drugs, efficacy at lower doses, potential recovery in both acute and chronic phases, and absence of mandatory hospitalization for the administration of these medicines [12].

NDs are conditions that, together with their predominance in contexts of poverty, still cooperate to survive the panorama of disparity, since they show strong obstacles against the development of targeted countries.

The present review discusses the formulation approaches currently being employed in the management of two important neglected diseases, namely, Leishmaniasis (LM) and Chagas’s disease (CD). It also discusses the classical drugs used for the treatment of these diseases, along with the pharmacological combinations available. An insight into the use of natural drugs has also been provided, followed by novel formulation strategies to incorporate classical drugs into new drug delivery systems (DDS). This review further discusses the role of contemporary formulations in improving the pharmacokinetic profile of the available drugs and to enhance their therapeutic performance [13]. 

## 2. Key Facts of Neglected Diseases: Leishmaniasis and Chagas’s Disease

According to the WHO’s classification, neglected diseases are divided into two categories: i) Preventive chemotherapy and transmission control (PCT), and ii) Innovative and intensified disease management (IDM). While the former is controlled by administering safe, effective and cost-effective drugs from time to time to vulnerable populations, the latter stands out as a considerable challenge and call for more focused efforts for generating innovative tools to control these diseases. These categories have also been referred to as ‘tool ready’ and ‘tool deficient’, respectively. LM and CD fall under the second category, not only by finding places in the list of the twenty NDs released by WHO, but also because they form a part of the Centre for Disease Control and Prevention plan of action. In 2012, the London declaration on NDs brought forth the goal of eradicating 10 of these destructive illnesses until 2020. It also highlighted the need to expand the eradication programs to avoid the spreading of the diseases and to cooperate in the field of research and development, aiming to create new therapeutic tools capable to destroy the parasites that are the origin of these epidemies [14,15,16]. 

According to the WHO’s reports, 700,000 to 1 million new cases of LM are recorded yearly, whereas 26,000 to 65,000 mortalities occur [17]. On the other hand, 6 to 7 million people are reported to be infected by CD globally [18]. Although CD is endemic in Latin America, it has been spreading to other countries (also identified as developed countries) by means of migration of the vector responsible for the disease transmission. The measures applied to control the dispersion include extensive actions to control the vector proliferation. Despite this, the need for vaccine development and discovery of novel antiprotozoal agents cannot be over emphasized. The dearth of pediatric formulations is another cause of concern [2]. Indeed, visceral LM and CD have been listed among NDs that have the highest mortality rate, while the drugs/formulations aimed to treat them are inadequate. The current strategy to manage NDs incorporates one or a combination of the following approaches: i) preventive chemotherapeutic measures; ii) intensive case management; iii) vector control procedures; iv) veterinary health; and v) innocuous water, correct sanitation and good hygiene habits [19].

## 3. The Diseases at a Glance

LM is a chronic pathology triggered by an intracellular protozoan belonging to the *Leishmania* family. It affects a high number of individuals worldwide and is endemic in several countries. In some developing countries, LM is a critical collective health problem. The disease is also endemic in southern Europe, with people moving to these regions being more likely to be infected. Due to a progressive occurrence of this illness, its relevance has expanded immensely. This increased incidence is, to some extent, associated with emigrants, displacements, war, housing in new regions, inadequate surveillance standards in some countries, and other situations that have contributed to an increase in cases of LM worldwide. There are several types of Leishmania, recognized according to the clinical manifestations they produce. Four are the outcome models of this disease: cutaneous LM (CL), which reaches the surface of the skin; mucocutaneous LM (MCL), affecting the surface of the skin and mucous membranes; diffuse cutaneous LM (DCL); and visceral LM (VL), which affects organs of the mononuclear phagocytic system [20]. The disease is transmitted by the bite of a phlebotomine insect—the vector. During the adult phase, this insect naturally adapts to a variety of environments and when it is in the larval phase, it grows in humid terrestrial milieus—with reduced clarity and abundant organic material. Dogs, marsupials and foxes are the natural reservoirs of the disease. The potential of these animals as a source of VL infection is, however, a subject of discussion. Nonetheless, there is no transmission from person-to-person, nor from animal-to-animal. 

In Portugal, as an example of a Southern European country, LM is a zoonosis caused by the parasite *Leishmania infantum* and the dog is the main host and the primary reservoir regarding the human visceral infection. The species of the vector are the *Phlebotomus perniciosus* and the *Phlebotomus ariasi*. Subtle changes in climate, as increasing temperatures, may lead to the exacerbation of the disease, spreading in both animals and humans, and increasing the prevalence of the pathology. Portugal has a predominance of LM in children, although this trend has been decreasing. Nevertheless, a marked increase in adults with human immunodeficiency virus /acquired immunodeficiency syndrome (HIV/AIDS) is significant. Although CL has a small incidence in Portugal, it is estimated that approximately 10 new cases are diagnosed each year. Moreover, canine LM has increased by approximately 20% in endemic areas (namely, great Lisbon and Algarve) [21,22,23].

CL is expressed in the human organism as an ulcer and usually heals on its own and generates a small scar [24]. The DCL is more complex, producing lesions that diffuse along the skin and with less capacity for recovery [25]. In the case of MCL type, skin wounds initially develop and may spread to the mucous membranes of the face; if inflammation occurs, it can particularly lead to erosion of the nasal cavities and the oral cavity [22]. In VL, skin lesions take approximately 2 to 8 months to propagate in parallel with severe inflammation responsible for lesions in liver and spleen, which can even lead to death if untreated [26]. Microscopic identification of parasites, predominantly the detection of amastigotes in tissue samples, polymerase chain reaction (PCR), serological tests, and immunofluorescence, among others, is of paramount importance [27].

CD, of which the etiologic agent is *Trypanosoma cruzi*, is one of the most important widely propagated NDs. CD is usually transmitted by means of vectors, responsible for approximately 80% of the contagious events, and occurs by the contact of humans with contaminated excrements of the insect *Triatoma*—the vector. This latter is infected after nourishing itself with some host present in the environment, further enabling the spread of the disease to another mammal, including man. The transfusion of blood, blood products by themselves or transplacental infection are other routes of spreading the disease, at any stage of development. However, the contamination of blood supply has so far not been the predominant form. Nowadays, the ingestion of food infected with *Trypanossoma cruzi* is the principal route of dissemination of this disease to humans. Oral transmission of CD has also unexpectedly emerged in non-endemic regions, where the vector appeared to be under control, due to food exposure to triatomine feces. Micro-epidemics occur according to this modus operandi, leading to critical public health situations [28,29].

After entry into the host, parasites infect the host cells and differentiate into reproductive amastigote forms. The acute phase of infection comprehends inflammatory reactions and common symptoms, such as fever and facial edema (which is common, particularly around the eyes). A rather extensive and asymptomatic period can follow, during which the detection of parasites is quite laborious. This stage can last for 10 years. In specific circumstances, a growing inflammatory lesion may occur as a reaction to residual parasites, sometimes fatal to the heart, esophagus, colon and other organs. Together with the symptomatic diagnosis and acute phase microscopy (while it is possible to locate trypomastigotes in the blood), the serological diagnosis of *Trypanossoma cruzi* antigen is usually performed to detect antibodies. PCR techniques, clinical trials, surveillance studies, xenodiagnosis and blood cultures may also be performed [30,31].

## 4. Pharmacological Interventions

### 4.1. Traditional Pharmacological Approaches

LM and CD are examples of NDs subjected to a reduced attention by the government and the pharmaceutical industry. In the last 15 years, the global pipeline of antiparasitic medicines was reduced down to 0.1% of the employed universal capital [32,33]. These two pathological conditions have similar parasite taxonomical origin, morphology and biochemical characteristics. The medicines currently used in the treatment of LM and CD have marked toxicity, inconstant effectiveness and require formalities for management or compliance with parenteral therapy regulations. CD has few drugs or treatments in clinical evolution. In contrast, for LM, there has been considerable progress with liposomal AmB (AmBisome^®^), miltefosine and paromomycin [34].

Nonetheless, research has so far been mostly inconclusive. It is critical to emphasize four particularities on the biology of *Leishmania*, especially on the activity of drugs:

- the intracellular location of the amastigote form;
- the distinct pharmacokinetic requirements to transport active substances in liver, spleen, bone marrow (on VL) or skin (on CL);
- the significant variations in drug susceptibility of the 17 genera of *Leishmania* responsible for LM in humans;
- the effect of immunosuppression linked to LM, which may reduce the efficacy of certain drugs.

The absence of a human vaccine against LM endorses chemotherapy as an election procedure in the treatment of LM and CD. Although there are several available chemotherapeutics against human LM, much of them rely on new formulations of old drugs [35]. The drug of election to handle all forms of LM has so far been pentavalent antimony. A wide range of LM patients in India, for instance, currently have a high degree of resistance to pentavalent antimony. Despite this, the therapeutic usefulness is kept in other areas of the world but with restricted license given the long treatment durability, the parenteral administration inevitability and the description of markedly mortal toxic effects (namely for the heart, liver and kidneys). In parallel with VL, some approaches are also restricted in the therapeutic of CL [36]. In the English-speaking states, the commercially available formulation is sodium stibogluconate (Pentostam^®^), whereas in France, Spain and Portugal, the therapeutic agent used is N-methylglucamine antimoniate (Glucantime^®^). Despite successive failures in therapy with pentavalent antimony, both medicines ((Pentostam^®^ and Glucantime^®^) have so far remained as the first-line drugs against all types of LM (despite several therapeutic failures with pentavalent antimony) due to their valuable effect at a lower price compared to second-line drugs [37,38].

AmB is a polyene antibiotic used as an antifungal, with a high action against promastigotes and amastigotes. This drug is used as a second option, since it presents several adverse reactions and, therefore, should only be used when antimony treatment is not effective. In India, this drug is the current therapy of choice, since pentavalent antimony has ceased to reveal activity [39]. AmB heads the therapeutic arsenal for the treatment of VL in a sequence of lipid-based formulations for the therapy of systemic mycoses in immunocompromised patients [40]. Of the suitable treatment for VL, only the liposomal formulation AmBisome^®^ is patented for VL and only in some parts of the world. After negotiations with the manufacturers, the cost of this product decreased, but several ampoules are still vital for single-course treatment and their stability in a small range of temperatures is controversial, as well as the potential risk of adverse reactions. Therefore, universal use of AmBisome^®^ for VL requires an extensive analysis in confined regions of the world [41,42].

Pentamidine is an aromatic diamine with marked activity as a substitute to antimony therapy. However, due to its intrinsic toxicity, its use is quite limited [43]. In previous years, the aminoglycoside paromomycin exhibited leishmanicidal effect. Later, some trials were very successful with this therapy and had the benefit of a reduced price in the market. Despite this, according to different healing responses in distinct locations, it is not used nowadays in monotherapy due to several inconclusiveness in published clinical literature [43]. In contrast, miltefosine was the primary oral treatment for VL with leishmanicidal effect. This substance has a 94% activity and the antiparasitic was exclusively subjected to phase-IV tests. Nevertheless, certain concerns on its application are raised, namely, the risk of malformations in the embryo, possible relapses after 28 days of treatment and limited patient cooperation, requiring additional care according to the drug resistance profile [44].

Sitamaquine (chemically, 8-aminoquinoin) is the single therapeutic agent designed for the treatment of VL and can be administrated orally, which is a considerable advantage for patient compliance. However, despite its relevant therapeutic effect, some adverse reactions have been reported, being vomiting, dyspepsia, cyanosis and nephrotic syndrome the most prominent. The mechanism of action of this drug is associated with the inhibition of the mobility, structure and development of the parasite. Nevertheless, more trials are still needed to understand the overall pharmacokinetics and pharmacodynamics of this drug [45,46]. Triazole and imidazole drugs, known as antifungal agents, also exhibit activity against some *Leishmania* species. They are however limited for subsequent trials due to their low water solubility [47].

Medicines currently applied in the treatment of VL and TL, such as Pentostam^®^, Glucantime^®^, pentamidine and amphotericin B, have no therapeutic effects if administered orally and require long-term parenteral administration. Additionally, these chemotherapeutic agents are expensive and have marked toxicity. Together, all these aspects lead to the minimal compliance or even withdrawal of patients from therapy. On the other hand, if the therapy is interrupted, it will impact enormously on the disease spread and on the appearance of drug resistant strains [48,49].

CD, a pathology identified over 100 years ago, has a long chronicle of successive efforts to therapeutically control its progress. Among these, compounds with questionable outcomes are listed, such as gentian violet (used as prophylactic in blood banks), nifurtimox (NF) and benznidazole (BNZ) [50,51]. NF and BNZ exhibit strong activity with some variability depending on the strain of the pathogen to be eliminated, the difference in sensitivity to treatment between patients (affected by age and geographical location), the clinical phase of the disease when these drugs are administered (with maximal effectiveness of both in the acute phase), dose, course of treatment, and side effects (such as weight loss, drowsiness, excitement) [52]. NF is a trypanocide drug, acting not only against circulating trypomastigotes but also against amastigotes. Even though it was forbidden for extended periods of time in clinical therapy, its use was increased after suspending the therapy with BNZ. Trypanocide activity, with a half-life in plasma of approximately 3 hours, was reduced thereby requiring continuous administration. The most frequent adverse effects impair the central (CNS) and peripheral nervous system (PNS), with gastrointestinal (GI) manifestations, such as nausea, vomiting, stomach pain and diarrhea [53]. BNZ exhibits activity against various *Trypanossoma cruzi* strains. It is given orally in adolescents. A recent formulation for pediatric administration has been proposed [54]. Adverse reactions have also been commonly reported, particularly hypersensitivity skin responses, bowel and neurologic manifestations. This therapeutic agent acts by three different mechanisms: trypanocidal effect, increased phagocytosis and parasite lysis by an IFN-Ɣ-dependent mechanism, and the inhibition of parasite growth by NADH-fumarate reductase blockage [12]. 

Due to the amplitude of these reactions, treatments with NF were forbidden in some southern countries. The reduction in the activity of (mono)therapy against *Trypanossoma cruzi*, combined with the inadequacy and considerable low income of workers in the countries affected by CD, highlights the need for new therapeutic agents. Other drugs act in vitro against *Trypanossoma cruzi* but, at present, only these two (BNZ and NF) are applied in human therapy. Adverse effects have a higher incidence in individuals over 50 years old, but the administration of these drugs is not advised in patients within this age range. The World Health Organization (WHO) provides these medicines free of charges. Both drugs are administered orally using a follow-up method to monitor patients and their administration is strictly forbidden during pregnancy, and conditions of hepatic and renal failure. Comparative trials have so far not been performed and, therefore, it is unknown to which one of the two drugs the adults infected with *Trypanossoma cruzi* present greater tolerance [55]. Some studies argue the formation of free radicals and/or electrolyte metabolites to explain the NF and BNZ mechanisms of action [56].

### 4.2. Pharmacological Combinations

The combination of drugs to treat LM has become a gradual procedure adopted because of the progressive absence of viable results with single-drug regimens. Therapeutic associations in VL bring several advantages. Indeed, the association of drugs belonging to different chemical groups allows the reduction in the time of treatment and in the integral amount of drug employed, which, in turn, leads to the reduction of toxicity, enhanced compliance and reduced individual burden. Additionally, the cost of the therapy is also reduced. Drug resistance is another problem that generates gaps in the treatment [57,58,59]. Parasite resistance to miltefosine due to mutations has also been studied, but the administration of this drug in VL has been reported for a limited period. Thus, combined therapy can contribute to delaying drug resistance and increase the half-life of drugs [60,61]. 

Such combinations can even optimize therapeutic efficacy in HIV-infected patients in whom monotherapy treatment has not been effective. Several assays have shown an increase in the leishmanicidal activity in a certain combination of drugs [62]. 

A formulation of paromomycin with sodium stibogluconate in VL treatment with positive responses in Sudan was evaluated [63]. The association of oral allopurinol with intravenous Pentostam^®^ was also applied against VL in Kenya. Although the therapy was initially successful, some redress occurred later. A study comparing a combination of allopurinol and antimony, with these two drugs separately, was carried out in the treatment of canine LM. The results showed that combined therapy of the two drugs offered better response than each one separately. Allopurinol was shown to inhibit CL expansion, but only when used as an adjuvant to pentavalent antimony. The combined treatment condensed the course of antimony therapy and long-term administration of allopurinol was satisfactorily accepted [64]. These results support combined chemotherapy as a viable solution in the treatment of CL. However, the responses are still very inconclusive and require additional clinical studies.

Imiquimod is an imidazoquinoline which, together with meglumine antimoniate, is an excellent alternative for the treatment of CL [65]. Nevertheless, a large part of the reported results is obtained from a small number of patients. Thus, more trials are needed to strengthen conclusions [66]. Another study assessed the lack of available combinations of drugs for VL, although coadministration of some leishmanicidal drugs was evaluated through experimental and preclinical toxicokinetic analyses however with no tangible results. Some trials demonstrated that a combination of AmBisome^®^ with paramomicin or miltefosine, as well as miltefosine with paromomycin, reduced the cost of treatment and was successful in the restraint of the pathology [40,67].

In CL, it was verified that azole antifungals, such as fluconazole and itraconazole, have some therapeutic effect. Further studies of adjuvant therapy with immunomodulators with recovery capacity, and a combination of bacillus Calmette-Guérin and antimony, were also performed. Here, once more, the combination of antimony plus another drug exhibited increased activity compared to each drug isolated [68]. 

All the studies carried out so far on CD revealed that an infallible pharmacological treatment was not yet achieved, especially for the chronic phase of the disease. Thus, one of the most beneficial options in CD therapeutics is combination therapy, especially the synergism between BNZ and other azole derivatives, such as itraconazole [69]. Nitric oxide administered together with BNZ has also been useful in preventing death through the reduction of parasitemia and inflammation in the heart [70].

### 4.3. New Drugs

The design of new drugs for CD is mainly hampered because patients who enter in an undetermined phase of disease development do not express symptoms and there is an enormous complexity to identify parasites. Despite this, several drugs are found in medical studies for this pathology. Inhibitors of lanosterol, notably posoconazole and E1224, have proved very promising. Both are available orally and are in the preliminary testing phase [69,71,72]. The cysteine-​ protease inhibitor has also shown activity in prototypes of chronic rodents and is in pre-clinical development. To avoid the marked adverse reactions of NF and BNZ used in CD, as well as the lack of pediatric formulation and its reduced activity in eliminating the parasite, clinical trials with antifungal triazoles (ravuconazol, voriconazole) are being carried out [73,74,75,76]. Yet, certain antifungals, such as ketoconazole and itraconazole, are not capable of inducing a complete parasitological recovery of this disease [77,78]. Posoconazole showed a promising response in the treatment of patients in both chronic and acute phases, especially in BNZ resistant strains, and is currently the candidate of choice. The most relevant benefits are mainly its marked activity and selectivity, therapeutic effect against resistant strains, greater tolerance and safety profile. The most significant constraints are the high associated amounts and high manufacturing costs [71,79].

The discovery of nitroreductase in *Trypanossoma cruzi* underlies the idea that the compounds have the potential to be activated by parasites rather than by host cells, reducing adverse reactions, and the ability to occur mutations, a concern associated with these compounds [80,81].

Regarding LM, and although VL is a lethal disease, no human vaccine that can be used has yet been developed, and the ones under development are directed to the less critical configuration of the pathology, which is the cutaneous form. Investigating self-resolving infections or spontaneously immune people provides important insight into the possible manufacture of these vaccines [82,83].

The immunization against *Leishmania* encompasses the inoculation with the species of parasites in a covered area to avoid the appearance of lesions in exposed regions of the body. However, some analyses have indicated a poorly effective defense against VL and a low probability of immunity to other vaccines [84,85]. Another assay was performed with leishmanolysin, a relevant virulence agent that could be employed as a vaccine against LM. As such, it has been observed that gp63 is the richest glycoprotein in *Leishmania*. Moreover, gp63 is linked to the defense against lysis caused by the complement system. Published studies ensure that this glycoprotein can activate cytokines involved in the Th1 response. In immunology, a vaccine should induce the Th1 response and reduce immunosuppressive conditions. Given this, it is estimated that gp63 or leishmanolysin is a relevant virulence factor in CD and is therefore a suitable candidate for vaccine design [86,87,88].

Drugs, such as pentamidine, have been used for a few dozen years in these two diseases. However, studies conducted to identify other therapeutic agents demonstrated a linkage with improved pharmacological and safety attributes than with pentamidine. Arylimidamide (DB766), given orally to rats, exhibited benefits against resistant strains, although with a narrow therapeutic window [89]. In another in vivo study using mice, the results revealed improved benefits with DB613A, which exhibited clinical effectiveness with half of the maximal inhibitory concentration in amastigote forms [90].

### 4.4. Herbal Treatments

Since ancient times, herbal, animal and mineral compounds have been used in classical medicine against human diseases. For centuries, these approaches have been the only available approaches to treat human diseases [91]. Today, in underdeveloped countries, approximately 80% of individuals are almost entirely dependent on this “natural medicine” approach to address their basic health needs [92]. Natural substances and/or extracts have received the attention of the pharmaceutical industry worldwide to develop new formulations with a potential therapeutic effect. Among the most analyzed natural substances, extracts of plants predominate in the origin of constituents with leishmanicidal effect [93]. Drugs identified from natural sources have been listed by numerous laboratories worldwide.

The main advantages of phytocomposites include the protection against toxic effects, the enhancement of the therapeutic effect, increased safety, increasing retention time, and defense against physical and chemical degradation [94]. 

Several plants were found to exhibit therapeutic activity against leishmania, such as *Kalanchoe pinnata*, *Plumbago scandens*, *Physalis angulata*, *Piper aduncum*, *Tabemaemontana australis*, and *Phyllanthus amarus* [95].

Alkaloids, as secondary metabolites, are particularly relevant in plants as a source of protection against various microorganisms and herbivores. These compounds are equally essential for man when applied to kill parasites. A countless number of alkaloids are described with noteworthy leishmanicidal activity, but without clinical results due to lack of clinical trials. The chemical structure of alkaloids with evidenced leishmanicidal activity is related to quinoline, indole, isoquinoline, naphthylisoquinoline, bisbenzylisoquinoline, estrogens, benzoquinolizidine, diterpenes, pyrrolidinium, acridone, β-carboline and marine sponge-derived terpenoids [96]. Table 2 lists the compounds with potential therapeutic value in the treatment of LM.

In recent decades, efforts have been made worldwide with the aim to find therapeutic solutions for CD with less adverse events for patients under treatment. The success encountered in this attempt depends on several factors: (i) the phase of the disease in which patients start the treatment (usually, it occurs predominantly at the later stage due to the asymptomatic acute phase of CD), (ii) the diversity of resistance profiles of parasites, (iii) the difficulty in the employment of the correct type of drug for individual patients (broad or narrow spectrum drugs) and, most importantly (iv) on the limited funding attributed to research groups devoted to the discovery of new targets and drugs.

Current data about the parasites, their life cycle and the known critical cell biomolecules required for the development of these plagues, drive researchers to look into the nature of potential therapeutic molecules as in loco microbiota of some flora and fauna develop mechanisms against these aggressors. Table 3 lists the compounds with potential therapeutic value in the treatment of CD.

## 5. New Drug Delivery Systems

A range of new drug delivery systems (DDS), such as liposomes, microemulsions, and micro/nanoparticles, proved to be very useful for the pharmaceutical industry. New liposome-based drug delivery systems are the most commonly used DDS due to their high biocompatibility, manufacture simplicity and chemical variability. They are biodegradable, biocompatible, non-immunogenic and highly versatile for research, analytical and therapeutic applications [110].

DDS are an additional alternative to the design of new drugs and combined therapy for VL. Such systems can preserve the medicine against the first pass metabolism upon oral administration and are also suitable for administration by other routes. DDS can overcome the limitations of reduced water solubility, in the same way as they allow the simultaneous transport of several drugs, thus facilitating the application of combination therapy [111].

The limitation of conventional therapy may be attributed to difficult and inadequate diagnosis, cases of drug resistance, inaccurate dosage, difficulties in adequately monitoring the dose, frequency of administration, poor patient compliance, lack of physical procedures to support the treatment, inadequate immune response of patients to therapy. Some other biopharmaceutical aspects govern the design of DDS including drug permeability through cell membranes, the mechanism of cell uptake (especially in the case of intracellular parasitic infections), stability, activity and kinetics in the target cell environment [112,113]. 

Liposomes and nanoparticles are especially viable against VL (depending on the drug they carry), because they are more easily captured by the mononuclear phagocytic system, which becomes an additional advantage in the treatment of this pathology. In fact, intraperitoneal (IP) and intravenous (IV) administration of liposomes was proven to be an excellent approach as it increased the accumulation of the drug-loaded liposomes in tissues with high concentration of macrophages, such as the spleen and liver—organs where *Leishmania* parasites are also present in vast quantities [114,115].

Liposomes are vesicles consisting of one or more phospholipid bilayers surrounding an aqueous core [116]. Some studies demonstrated that liposomes-encapsulated antimony had a 700-fold therapeutic effect against canine LM when compared to the non-encapsulated form [117]. Another example of a drug developed in this context was liposomal AmB (AmBisome^®^), which was shown to be 350 to 750-fold more effective than non-encapsulated meglumine antimonate and AmB. The latter formulation decreased the toxic effects at a cellular level with improved stability and activity against VL [118]. Currently, this is the only DDS-based product authorized for LM therapy. The example of liposomal AmB clearly shows that the design of DDS can substantially improve the performance of loaded drugs. 

Niosomes are vesicles consisting of non-ionic surfactants and cholesterol with similar characteristics as liposomes. In order to be cost-effective, safe and allow optimizing the therapeutic effect against LM, the surfactants require a thorough selection process [119]. AmB, in combination with selenium, has been recently formulated in niosomes [120]. The combined noisome therapy exhibited higher inhibitory effect on the promastigote and amastigote forms of Leishmania tropica L. than the non-loaded form. The loaded form significantly decreased the levels of interleukin-10, while the levels of IL-12 and metacasoase (as Th-1 activator) significantly increased.

Solid lipid nanoparticles (SLN) consist of nanoscaled DDS with the matrix composed of solid lipids at room and body temperatures [121,122]. These systems are considered highly safe and simple to manufacture in a substantial extent. They also allow controlled release of drugs and immediate removal by the liver and spleen [123]. Although the trials still do not allow draw conclusions, SLN may be proposed to promote leishmanicidal and antimalarial effects [124]. Enhanced drug absorption at the hepatic level has been reported, as well as the increase in the leishmanicidal therapeutic effect using mannose-coated AmB lipid nanospheres [125].

Other patented AmB lipid-based DDS for therapeutic use are the colloidal dispersions of AmB (Amphocil^®^) and the lipid complexes of AmB (Amphotec^®^). These DDS also decreased the toxic effects and increased the therapeutic index of loaded drugs [126]. AmBisome^®^ is applied for the treatment of parenteral VL, already having generics in the market [127]. 

Otheres class of DDS are the polymeric nanoparticles, which can be further divided into nanospheres, when the matrix is composed of chains of continuous interconnected polymers, and nanocapsules—resulting from the surrounding of oil or water nanoparticles by a thin polymer coating. Both types of nanoparticles allow optimizing the release of the drug [128]. 

Primaquine-loaded nanoparticles were tested, with a 21-fold higher response in terms of drug release and with fewer side effects [129]. Similar results were observed with the use of nanoparticles containing AmB [130]. Pentamidine nanoparticles were also evaluated, and the response was 25-fold higher in terms of drug release and with less side effects [131].As previously mentioned, with the exception of miltefosine and sitamaquine, leishmanicidal drugs have a very low oral bioavailability. This aspect is the result of a highly frequent enzymatic decomposition, reduced membrane permeation at the level of the intestine and, more relevant, the reduced solubility in water. To overcome these limitations, some alternatives may be adopted, such as oral DDS, aimed at increasing drug solubility, improving its half-life and absorption profile [132].

### 5.1. Targeting the Values of Apparent Solubility

DDS increase the contact area of the drug with the absorption tissue and facilitate decomposition in the GI tract in case of oral administration. Polymeric nanoparticles and nanosuspensions are some examples [133]. A study conducted with AmB, formulated in nanocapsules containing poly(lactic-co-glycolic acid) (PLGA) nanocapsules, found it to be 8-fold superior to Fungizone^®^, both in oral bioavailability as well as in antifungal activity [134]. Nanosuspensions are composed of the drug crystals surrounded by a surfactant layer, generally in the form of an aqueous dispersion. Some antiparasitic agents were analyzed in nanosuspensions and successfully evaluated by in vitro and in vivo assays against *Leishmania* [135]. Particularly noteworthy is the nanosuspension of AmB having a particle size of approximately 530 nm, obtained by high pressure homogenization (HPH) with a set of surfactants: pluronic F68, Tween 80 and sodium cholate. This formulation was more effective against VL after oral administration in the rat, than AmBisome^®^, Fungizone^®^ and micronized AmB administered under the same conditions, which showed no activity [136].

#### 5.1.1. Cyclodextrin Complexes

Another way of increasing drug solubility to assist oral absorption is using cyclodextrins. Cyclodextrins are hydrophilic cyclic oligosaccharides which possess a hydrophilic extrinsic space and a central lipophilic concavity employed as promoters of drug absorption upon delivery. Generally, the hydrophobic drugs are encapsulated in the central opening, increasing the drug water solubility [137]. Through a sequence of tests, it has been proved that meglumine antimoniate complexed with cyclodextrin yielded an improved oral bioavailability [138].

#### 5.1.2. Modification in the Absorption Behavior

Lipid-based DDS (e.g., micro/nanoemulsions, mixed micelles and lipid-based nanoparticles [139,140,141]) have a high ability to improve drug bioavailability. Using lipids as additives, they improve drug absorption in the gut and may be also metabolized by the gastrointestinal (GI) enzymes. 

An emulsion is a dispersion of small droplets of a liquid in a second liquid in which the first is not miscible and usually requires one or more surfactants to balance the interface. For nanoemulsions, the droplets are within the nanometer range [142]. The production of an AmB-loaded oil-in-water (O/W) microemulsion showed ability to increase drug dissolution by 1000-fold with a reduction in toxic effects on cells [143]. Also, another test performed with a self-emulsifying formulation of AmB with mono- and diglycerides plus vitamin E (stabilizer) proved to be stable in the intestinal fluid with the AmB concentration remaining constant for 6 hours and with improved oral bioavailability [144]. Mixed micelles with amphiphilic compounds are able to reproduce the physiological systems where the lipids from the food are transformed into mixed micelles constituted by bile salts [145]. AmB-loaded micelles composed of phospholipids and bile acids improved the absorption of the drug in a prototype of intestinal loop perfusion in rats, although not tested in the healthy animal [146]. Lipid-based nanoparticles have benefits compared to other oral DDS, since they possess good physical stability and simplicity to obtain hydrophobic drugs. Besides SLN, cubosomes and nanocochleats have also been described [147]. Their effects were studied against LM, and an AmB-based formulation was developed exclusively for oral administration. The study reported an increased bioavailability response, as well as increased half-life through controlled drug release [148].

Cubosomes are crystalline lipid structures with cubic symmetry formed by amphiphilic molecules which contain two alternating nonhomogeneous aqueous phases delimited by lipid bilayers. They have a great ability to encapsulate hydrophilic active substances, protecting them against the external environment. Consequently, they are suitable for oral administration [149]. A study was carried out with cubosomes containing AmB, but there was no biological response [150]. Nanocochleats are cigar-shaped nanostructures consisting of negatively charged lipid bilayers, attached to a divalent cation, usually calcium. The benefits of this type of DDS are essentially the safety they provide to the drug against the GI medium, defense against oxidation and preservation of the morphology after lyophilization, which allows its storage for extended periods at room temperature [151]. AmB-loaded nanocochleats showed improved oral absorption and antifungal activity relative to free AmB [152].

#### 5.1.3. Increase in Resistance Time

Another alternative that aims to promote the oral bioavailability of drugs is the increase in the drug residence time at the absorption site. When binding the intestinal wall, the DDS increase the local concentration gradient of the drug at the absorption site, by prolonged contact of the DDS with the mucus, increasing the portion of drug capable of being absorbed. Moreover, the DDS may include additives to promote diffusion through the mucosal epithelium and thus favoring drug permeation [153]. 

Chitosan is an interesting polysaccharide, combining bio-adhesion with absorption-enhancing characteristics. Chitosan is a natural polysaccharide composed of glucosamine and N-acetylglucosamine which is obtained from chitin of crustaceans. This compound may further function as an absorption promoter through the intestinal epithelium, due to its capacity to strengthen the diffusion of mucoadhesive systems. Recently, a preparation of chitosan nanoparticles demonstrated a reduction in toxic effects via IV administration and good leishmanicidal activity in a rat model [154].

The main challenge of CD pharmacotherapy is reaching the overall disseminated intracellular parasites. Intracellular bacterial infections caused by protozoa present physical barriers that hinder the arrival of significant portions of the drug to the target site. However, BNZ and NF exhibit a high volume of distribution (Vd) and with a poor access to intracellular targets leading to high plasma concentrations and toxic effects. The ability to alter the surface of target cells and tissues as well as phagocytosis offers DDS the ability to circumvent these natural barriers and transport the drugs in an extraordinarily small volume, reducing their concentration in circulating fluids and non-target tissues [155,156]. Therefore, the efficacy of liposomes against trypanosomiasis or CD has been investigated to a much lower extent than LM. This state possibly results from the widespread positioning of the parasites in cells of the mononuclear phagocytic system (MPS) making them difficult to reach with the use of liposomes, which are defined by agglomerating in MPS after IV injection [157].

Nakae et al., in two studies, evaluated the efficacy of stearylamine-liposomes with positive charge (drug-free) against *Trypanossoma cruzi*. More interestingly, positively charged liposomes with phosphatidylcholine and stearylamine demonstrated the ability to destroy *Trypanossoma cruzi* in a brief time (30 minutes), at a moderately reduced lipid dose (10 µM) and without toxicity to erythrocytes. In turn, phosphatidylcholine and stearylamine tested alone did not reveal any activity against parasites, reinforcing the idea that the association of both in a liposome is of extreme relevance for antiparasitic activity [158,159].

Another study, conducted by Morilla et al., with liposomes encapsulated with BNZ, concluded that although their concentration in liver and blood increased, the efficacy of this formulation in the treatment of CD was not feasible, since liposomes were not capable to make BNZ reach the cytoplasm, where the parasite is usually located [160]. Morilla et al., in another study, evaluated the usefulness of pH-sensitive liposomes incorporated with etanidazole (ETZ) to promote the arrival of this type of liposome into the cytoplasm. This attempt, unlike ETZ alone which did not show any activity, resulted in approximately 72% of parasites dead and parasitemia reduction. Nonetheless, this association did not eliminate the parasite, but contributed to the advancement of upcoming studies with liposomes pH-sensitive [161].

Yardley et al. related the activity of AmB in vitro and in vivo, namely in the products AmBisome^®^, Amphocil^®^, Abelect^®^, for the treatment of CD. Although Amphocil^®^ proved to be more efficient in vitro, Ambisome^®^ demonstrated superior activity in reducing parasitemia in vivo than the other formulations [162].

Gonzalez-Martin et al. pointed out the utility of NF-loaded polyethylcyanoacrylate nanospheres targeted against trypomastigotes and intracellular amastigotes with IC_50_ results demonstrating a 21-fold lower proliferation in comparison with non-encapsulated drug treatments [163]. The same authors also demonstrated the in vitro activity of allopurinol-loaded polyethylcyanoacrylate nanospheres against *Trypanossoma cruzi* epimastigotes, with a response twice as effective in comparison with the free drug. However, to be validated, the studies require in vivo confirmation of the nanospheres efficacy [164]. Molina et al. performed an experiment with DO870 nanoparticles (PEG and lactic acid) injected intravenously in a mouse model infected with *Trypanossoma cruzi* [165]. The formulation led to the cure of trypanosomiasis with a dose-dependent activity. Regardless of the few advances achieved through these studies, they are expected to contribute to original approaches as they are still very inconclusive and require further research.

In the last decade, the progress of nanoscience has similarly been applied to drugs of natural origin, producing promising and useful tools in the treatment of several pathologies, including LM. Nanometric-scale formulations prepared with products derived from medicinal plants and their leishmanicidal activity are reported below, with liposomes, niosomes, and nanoparticles as the main addressed systems. Saponins (liposomes) and alkaloids (nanoparticles) are the essential plant components used in these frameworks. A considerable effectiveness of flavonoids against *Trypanossoma* and *Leishmania* species has been described by means of an assay using quercetin. Following studies tested quercetin formulated in niosomes, liposomes and microspheres, with overall experimental hypothesis demonstrating reduced parasitemia relatively to the free drug, from which can be deducted that encapsulated quercetin is more effective in containing LM [166,167].

The loading of terpenoids in micro/nanoparticles also exhibited therapeutic activity. Yet, effective drugs against LM are still a demand, while most products are characterized by their low solubility compromising the drug bioavailability. Nanoparticles loaded with andrografolide, a diterpenoid extracted from the *Andrographis paniculata*, displayed strong leishmanicidal activity [168]. It was also proved that particle size is a relevant factor for drug transport efficiency. In fact, nanoparticles with smaller than 200 nm have been linked to increased phagocytosis by macrophages infected with *Leishmania* [169,170].

Another study with multiparticle systems, including niosomes, microspheres, liposomes and nanoparticles, improved leishmanicidal activity with the use of Bacosaponine-C (extracted from *Bacopa monnieri*), by reducing the parasitic load on the spleen of hamsters compared to animals tested with free drug. With the same compound amount, the best activity, in diminishing order, was observed for: nanocapsules > niosomes > liposomes > microspheres [171]. Despite the promising results, the use of saponins is restricted as a consequence of their high toxicity.

Another compound with a visible effect against LM is amarogentine (obtained from *Swertiachirata*). The liposomal and niosomal preparations of this molecule decreased the parasite incidence in the spleen by 69 and 90%, respectively, while the free drug in the same amount obtained an outcome of 39% [172]. In turn, the piperine alkaloid (extracted from the *Piper nigrum*), had a strong effect against both VL and TL. The loading of this substance in liposomes reduced the parasitemia by approximately 70%, whereas, in its free form (in addition to being less effective), it also demonstrated greater toxicity with the top results obtained with piperine nanospheres [173]. Harmine (isolated from Syrian *Arruda*) manifested leishmanicidal activity in vitro and, when incorporated into liposomes, niosomes, or nanoparticles, this alkaloid was able to reduce parasitemia in the spleen of infected animals. However, the harmine action mechanism against *Leishmania* is poorly elucidated, thus requiring further study [174].

#### 5.1.4. Programmed Cell Death

Programmed cell death (PCD) is described as a strictly controlled event allowing organisms to reject cells, thereby preventing inflammation usually occurring in injured surrounding cells. This process was described for the first time in the 1970s and, since then, it is being used to describe cancer and degenerative disorders. PCD allows a distinct type of cell death depending on the organism—mostly eukaryotic cells. In recent years, PCD has also been described in prokaryotes [175].

*Leishmania* and *Trypanosoma cruzi* are unicellular protozoa, being the etiological cause of LM and CD, respectively. Their life cycle development requires several stages, which entail various hosts until they infect humans and proliferate. Current literature highlights the importance of PCD in the differentiation of infectious and non-infectious forms of these parasites. The infectious forms do not divide while the non-infectious forms have an undefined track. Some phenotypical features of these protozoa are similar to eukaryotes such as the loss of the depolarization of mitochondria membrane, exposure of phosphatidylserine to the outer aspect of the cell membrane, breakage of DNA structure, cytochrome C release from the mitochondria and enhanced proteolytic activity. A similar expression of molecules in the transduction signals of PCD in eukaryotes was also demonstrated for these parasites. Both in vitro (using distinct harsh conditions as AmB or H_2_O_2_) and in vivo (using *L. donovani* amastigotes extracted from macrophages of patients treated for LM) assays detected signals of evidence of PCD [176]. 

Despite the prediction of similar mechanisms on PCD, researchers now have indication that this occurrence of PCD in prokaryotes is a distinct event from apoptotic conditions in mammalian organisms. 

The reason behind the presence of such forms of PCD is still unclear but is probably linked to the required cell machineries in these organisms to evolve and resist to host defenses. Regarding *Leishmania*, the transmittable form of the parasite is the metacyclic form, which develops from procyclic promastigotes inside the vector. The first form of the parasite is capable of infecting the hosts, evolving to amastigotes and initiating an infectious process. The process of differentiation does not occur in every single cell and, because of that, PCD may possibly be a process to select only the viable cells to perpetuate the species. This is achievable as the non-infectious parasites would be eliminated and, so, only infectious forms would use the nutrients required to survive and proliferate. After infecting the organism, the parasite amastigote form reproduces slowly over the years and releases the infectious form to the bloodstream but without triggering an inflammatory process. To achieve such a status, PCD may have a prominent role here by allowing phagocytes, namely macrophages, to be infected by T-lymphocytes undergoing apoptosis, rendering a refractory reply of macrophages to cytokines and yielding parasite survival. As both *T. cruzi* and *L. donovani* can undergo PCD, the halted immune response can be achieved, and parasite replication allowed. This fact is of topmost importance as a criterion for the development of new therapeutic strategies regarding the inhibition of key molecules in this event, which might contribute to future development in LM and CD therapeutics [177].

## 6. Conclusions

Chemotherapy is still the strategy of choice to eradicate NDs. This approach assists not only as a means of intervention and reestablishment of people infected by such pathologies, but also for health protection and the set-up of surveillance services. Pharmacovigilance, although still largely neglected, is extremely important and should be considered by professionals and health systems, especially in these scenarios, where knowledge about patients and transmission courses is relevant. The drugs used in ND therapy are old-fashioned in their design, possess side effects and present a high resistance profile, resulting in the need for the development of new drugs that present bioequivalence in vivo. Other important measures, which should receive further attention from governments, include reinforcements of the inhabitant’s education in these endemic areas, improving rudimentary hygiene, sanitation and monetary supplies. One of the limitations of pharmacological treatment is the failure to adopt some of these measures, leading to the spread of these infections. The only two drugs currently used in the treatment of CD are strictly active against the acute phase of the disease but not against the chronic. Furthermore, the adverse reactions arising from the administration of such molecules are harmful and are responsible for the poor patient compliance to therapeutics. Concerning LM, despite the progress already made in the knowledge of the parasite, the therapy still fails due to the enormous diversity of existing species with diverse characteristics. In this way, the lack of control of these pathologies may lead to the exacerbation of other illnesses, as is the case of HIV. In this sense, there is a vital need to develop new therapeutic systems that aim to overcome these limitations and improve the transport of the drugs to the target site, assuring an increase in therapeutic efficacy. One of the main objectives of new DDS is to design formulations that can be administered orally. DDS are extremely useful for poorly soluble drugs in water, as in the case of amphotericin B because they are formulated as dispersions, facilitating drug dissolution. These systems also have high amount of lipids, which contribute to drug absorption and longer residence time in the GI tract. Another interesting strategy is pharmaceutical research in the field of medicinal plant-derived compounds. The combination of phytochemistry with nanotechnology aims to create new possibilities on the screening of new antiparasitic drugs, since these preparations can enhance the activity of natural compounds. This is a field enriched in possibilities. However, most of the studies are still limited to basic research due to a lack of funding.

## Figures and Tables

**Table 1:** Description of the top 13 neglected diseases (NDs) according to their prevalence.

Pathology	World Prevalence (in Millions)	Risk Population (in Millions)	Prevalence Regions
*Ascariasis*	807	4200	South and East Asia, Pacific Islands, Africa
			Sub-Saharan Africa, India, China, Latin America and the Caribbean
*Trichuriasis*	604	3200	South and East Asia, Pacific Islands, Africa
			Sub-Saharan Africa, India, Latin America and the Caribbean
*Ancylostomiasis*	576	3200	South and East Asia, Pacific Islands, Africa
			Sub-Saharan Africa, India, Latin America and the Caribbean
*Schistosomiasis*	207	779	Sub-Saharan Africa, Latin America and the Caribbean
*Filariasis*	120	1300	South and East Asia, Pacific Islands, Africa
			Sub-Saharan Africa
*Trachoma*	84	590	Middle East and North Africa, Sub-Saharan Africa
*Onchocerciasis*	37	90	Sub-Saharan Africa, Latin America and the Caribbean
*Leishmaniasis*	12	350	South Asia, Sub-Saharan Africa, India, America
			Latin America and the Caribbean
*Chagas’s Disease*	8–9	25	Latin America and the Caribbean
*Hansen’s Disease*	0.4	-	Sub-Saharan Africa, India, Latin America and the Caribbean
*African Trypanosomiasis*	0.3	60	Sub-Saharan Africa
*Dracunculiasis*	0.01	-	Sub-Saharan Africa
*Buruli ulcer*	-	-	Sub-Saharan Africa

**Table 2:** Description of the compounds with potential therapeutic value in the treatment of Leishmaniasis (LM) [97,98,99,100,101,102,103,104].

Compound	Natural Sources	Targeted Parasite	Classes of Compounds
Renieramycin A	*Neopetrosia*	*L. amazonensis*	Alkaloids
Cyclic peroxide	*Plakortis aff angulospiculatus*	*L. mexicana*	-
Valinomycin	*Streptomycers spp.*	*L. major* promastigotes	-
Agelasine D	*Agelas nakamurai*	*L. infantum*	-
Diphyllin	*Haplophyllum bucharicum*	*L. infantum* intracelular amastigotes and promastigotes	Lignans
10-deacetylbaccatin III	*Taxus baccata*	*L. donovani* amastigotes	Taxoids
Linalool	*Croton cajuçara*	*L. amazonensis* promastigotes and intracellular amastigotes	Terpenes (mono)
7-hydroxy-12-methoxy-20-nor-abieta-1,5(10),7,9,12-pentaen-6,14-dione	*Salvia cilicica*	*L. donovani* and *L. major* intracellular amastigotes	Terpenes
Abieta-8,12-dien-11,14-dione			
-	*Lophanthera lactescens*	*L. amazonensis* amastigotes	Triterpenes (nor-)
Isoiguesterin	*Salacia madagascariensis*	*L. donovani* and *L. mexicana*	Terpenes
20-epi-isoiguesterinol			
8-epixanthatin 1_,5_-epoxide	*Xanthium brasilicum Vell*	*L. donovani*	Terpenes (lactone sesqui-)
Elephantopin	*Elephantopus mollis*	*L. major* extracellular promastigotes	Terpenes (lactone sesqui-)
2-deethoxy-2_-methoxyphantomolin			
Psilostachyin	*Ambrosia tenuifolia*	*Leishmaniaspp.* promastigotes	Terpenes (lactone sesqui-)
Simalikalactone D	*Simaba orinocensis*	*L. donovani* promastigotes	Terpenoids (Decanortri-)
Acetylvismione D	*Psorospermum glaberrimum*	*L. donovani*	Anthranoids
Dicentrinone	*Duguetia furfuracea*	*L. braziliensis* promastigotes	Alkaloids
Jatrogrossidione	*Jatropha grossidentata*	*Leishmaniaspp.* amastigotes	Terpenes (di-)
Jatrophone	*Jatropha isabellii*	*L. amazonensis*	Terpenes (di-)
Oleanolic acid	*Salvia cilicica*	*L. donovani* and *L. major* promastigotes and amastigotes	Terpenes (tri-)
Ursolic acid			
Luteolin	*-*	*Leishmania* *spp.*	Flavonoids
Quercetin			
6,7-dihydroneridienone	*Pentalinon andrieuxii*	*L. mexicana*	Sterols
Licochalcone A	*Glycyrrhiza* *spp*	*L. major* and *L. donovani* promastigotes and amastigotes	Chalcone (oxygenated)
20,60-dihydroxy-40-methoxy-chalcone	*Piper aduncum*	*Leishmania spp*.	Chalcones derivatives
-	-	*Leishmania spp*. promastigotes	Aurones
-	*C. brasiliense*	*L. amazonensis*	Coumarins
Casuarinin	*Punica granatum* *,* *Casuarina* *, and* *Stachyurus* *species*	*L. donovani*	Tannins
Amarogentin	*Swertia chirata*	*L. donovani*	Iridoids (glycoside seco-)
Plumbagin	*Pera benensis*	*L. donovani* promastigotes and intracellular amastigotes and *L. amazonensis* amastigotes and L. braziliensis and L. venezuelensis	Naphthoquinones
8,80-biplumbagin			
Burmanin A	*Diospyros burmanica*	*L. major*	Naphthoquinones
Burmanin B			
Burmanin C			
Pendulone	*Miconia lepidota*	*L. donovani*	Quinones
Primin			
Chimanine B	*Galipea longiflora*	*L. amazonensis*	Alkaloids (quinoline derivatives)
Chimanine D			
Cephaeline	*Psychotria klugii*	*L. donovani*	Alkaloids
Isocephaeline			
Klugine			
Harmine	*Peganum harmala*	*Leishmania spp.*	Alkaloids
Maesabalides III	*Maesa balansae*	*L. infantum* intracellular amastigotes	Saponins
Maesabalides IV			
Racemoside A	*Asparagus racemosus*	*L. donovani* amastigotes	Saponins
α- and β-Hederine	*Hedera helix*	*L. infantum* promastigotes and intracellular amastigotes	Saponins
Hederacholchiside A1			
α-bisabolol	*Matricaria recutita, Matricaria chamomilla and Vanillosmopsis arborea*	*L. amazonensis*	Sesquiterpenes
Undeca-2*E*,4*E*-dien-8,10-diynoic acid isopentylamide	*Anacyclus pyrethrum*	*L. donavani*	Alkamides
Tetradeca-2*E*,4*E*,12Z-trien-8,10-diynoic acid isobutylamide			
Deca-2*E*,4*E*,9-trienoic acid isobutylamide			
5-methyl-2,2′:5′,2″-terthiophene	*Porophyllum ruderale*	*L. amazonensis* promastigotes and amastigotes	Thiophene derivatives
5′-methyl-[5-(4-acetoxy-1-butynyl)]-2,2′-bithiophene			
Caffeic acid	*P. carolinensis, P. rosea* *and P. odorata*	*L. amazonensis* promastigotes and intracellular amastigotes	-
Chlorogenic acid			
Ferulic acid			
Quercetin			
Rosmarinic acid			
-	*Artemisia absinthium* *L.* *Artemisia dracunculus* *L.* *Artemisia seiberi* *L.* *Artemisia ludoviciana* *Nutt* *Artemisia abyssinica* *Sch.Bip*	*L. amazonensis* *L. aethiopica* and *L. donovani*	-
-	*Aphelandra scabra* *(leaves),* *Byrsonima bucidaefolia* *(bark),* *Byrsonima crassifolia* *(bark),* *Clusia* *fl* *ava* *(leaves),* *Cupania dentata* *(bark),* *Diphysa carthagenensis* *(leaves),* *Dorstenia contrajerva* *(complete plant),* *Milleria quinque* *fl* *ora* *(root),* *Tridax procumbens* *(complete plant), and* *Vitex gaumeri* *(bark)*	*L. mexicana* promastigotes	-
-	*Urechites andrieuxii; Colubrina greggii* *,* *Dorstenia contrajerva* *, and* *Tridax procumbens*	*L. aethiopica* promastigotes	-
3(S)-16,17-didehydrofalcarinol or oxylipin	*T. procumbens*	*L. mexicana* promastigotes and intracellular amastigotes	-
Cholest-5,20,24-trien-3β-ol	*U. andrieuxii*	*L. braziliensis* and *L. amazonensis* and *L. donovani* and *L. mexicana* promastigotes and amastigotes	-
6,7-dihydroneridienone			
Methylcholest-4-24(28)-dien-3-one, cholest-4-en-3-one			
Pentalinosterol			
Neridienone			
Diospyrin	*Euclea natalensis*	*L. donovani* promastigotes	-
Racemoside A	*Asparagus racemosus*	*L. donovani* promastigotes and amastigotes	Saponins
Amarogentin	*Swertia chirata*	*L. donovani*	Iridoids (glucoseco-)
Mesabalide III	*Maesa balansae*	*L. infantum* intracellular amastigotes	Saponins
Mesabalide VI			
Crocaudatol	*Croton caudatus*	*L. donovani* promastigotes and intracellular amastigotes	Terpenes
Crotocaudin			
Crotoncaudatin			
Isocrotocaudin			
Glycyrrhizic acid	*Glycyrrhiza glabra* *A. indica*	*L. donovani* intracellular amastigotes	-
2-phenylquinoline	*Angostura longiflora* *(* *Krause* *)* *Kallunki*	*L. braziliensis*	Alkaloids
6α,7α,15β,16β,24-pentacetoxy-22α-carbometoxy-21β,22β-epoxy-18β-hydroxy-27,30-bisnor-3,4-secofriedela-1,20(29)-dien-3,4 R-olide	*Lophanthera lactescens* *Ducke*	*Leishmania spp.* intracellular amastigotes	Triterpenes (nor-)
Piperine	*Piper nigrum*	*L. amazonensis* promastigotes and amastigotes	Alkaloids
Phenylamide			
β-pinene	*Artemisia campestris*	*L. infantum* promastigotes	-
Camphor	*Artemisia herba-alba*	*L. infantum* promastigotes	-
Camphor	*Artemisia annua*	*L. donovani* promastigotes and amastigotes	-
β-caryophyllene			
Buchtienin	*Kopsia grif* *fi* *thii*	*L. donovani* promastigotes	Alkaloids (indole)
Harmane			
Pleiocarpin			
Ramiflorines A and B	*Ampelocera edentula*	*L. braziliensis* and L. *amazonensis*, and *L. donovani* promastigotes	Alkaloids
2α,3β-dihydroxyursan-12-in-28-oic acid	*Pourouma guianensis*	*L. amazonensis* promastigotes and intracellular amastigotes	-
2α,3β-dihydroxyolean-12-in-oic acid			
Oleanolic acid			
Ursolic acid			

**Table 3:** Description of the compounds with potential therapeutic value in the treatment of Chagas’s disease (CD) [101,105,106,107,108,109].

Compound	Natural Sources	Targeted Parasite	Classes of Compounds
Helenalin	*Arnica spp.* and *Inula* *spp.*	*T. brucei rhodesiense* trypomastigotes	Terpenes (sesquiterpene lactones)
Mexicanin I			
Parthenolide	*Saussurea costus*	*T. brucei rhodesiense*	Terpenes (sesquiterpene lactones)
Primin	*Miconia lepidota*	*T. brucei rhodesiense*	Quinones
7,8-dihydroxyflavone	-	*T. brucei rhodesiense*	Flavones
Quercetagetin			Flavonols
Demethylpraecansone B	*Tephrosia aequilata*	*T. brucei rhodesiense*	Chalcones
Praecansone B			
Justicidin B	-	*T. brucei rhodesiense*	Lignans (arylnaphthalide)
Piscatorin			
Cissampeloflavone	*Cissampelos pareira*	*T. brucei rhodesiense*	Chalcones (flavone-chalcone dimer)
Lepadins D	*Didemnum spp.*	*T. brucei rhodesiense* trypomastigotes	Quinolines (decahydroquinoline)
Fascaplysin	*Hyrtios erecta*	*T. brucei rhodesiense*	Alkaloids (quaternary indole)
Ascididemin	-	*T. brucei rhodesiense*	Alkaloids (pyridoacridone)
2-bromoascididemin			
Manadoperoxide B	*Plakortis cfr. Lita*	*T. brucei rhodesiense*	Peroxide derivatives
Manadoperoxide I			
Manadoperoxide K			
Pandaroside G methyl ester	*Pandaros acanthifolium*	*T. brucei rhodesiense*	Steroids (saponins)
Dibromopalau’amine	*Axinella verrucosa*	*T. brucei rhodesiense*	Oroidins (dimer)
Brassicasterol	*Chondrosia reniformis* and *Tethya rubra* and *Tethya ignis* and *Mycale angulosa* and *Dysidea avara*	*T. cruzi* epimastigotes	Steroids (sterols)
*β*-sitosterol			
Stigmasterol			
	*Rhodnius prolixus*	*T. rangelii*	Lectins
Quercetin	*Guazuma ulmifolia*	*T. cruzi*	Flavones
Naringenin	-	*T. cruzi* trypomastigotes and amastigotes	Flavanones
Sakuranetin			
Borneol	*Croton pedicellatus* and *Croton leptostachyus*	*T. cruzi*	-
γ-terpinene			
*p*-cymene			
*trans*-caryophyllene			
Germacrene D			
Artemisinin	*A. absinthium* and *A. annua*	*T. cruzi*	-
*α*-thujone			
*β*-thujone			
sabinene			
*β*-pinene			
Myrcene			
*trans*-sabinyl acetate			
1,8-cineole			
Linalool			
*cis*-epoxyocimene			
Artemisiaketone			
Camphor			
Bornyl acetate			
Myrtenol			
Chrysanthenyl acetate hydrocarbon monoterpenes			
Sesquiterpene lactones			
Dihydrochamazulene			
*trans*-caryophyllene			
4-hydroxy-3-tetraprenylphenylacetic acid	*Spongiaspp.* and *Ircinia* *spp*	*T.b. rhodesiense*	Terpenes (furanoterpenes and meroterpenes and di- and tri-terpenes)
11β-acetoxyspongi-12-en-16-one			
Demethylfurospongin-4			
24-ethyl-cholest-5α-7-en-3-β-ol	*Agelas oroides*	*T.b. rhodesiense*	Steroids (sterol)
Pandaroside G	*Pandaros acanthifolium*	*T.b. brucei* and *T. cruzi*	Steroids (saponins)
Acanthifolioside E	-	*T.b. rhodesiense* and *T. cruzi*	Steroids (saponins) and Terpenes (acanthifolisides)
Acanthifolioside F methyl ester			
Plakortide P	*Plakortis angulospiculatus*	*T. cruzi*	Polyketide endoperoxides
11,12-didehydro-13-oxo-plakortide Q	*Plakortis* sp	*T.b. brucei*	-
10-carboxy-11,12,13, 14-tetranor-plakortide Q			
Manadoperoxides B	*Plakortis* cfr. *lita*	*T.b. rhodesiense*	-
Manadoperoxide G			
Peroxyplakoric ester B3			
Tetronic acid-containing tetromycin B	*Axinella polypoides*	*T.b. brucei*	-
Tetromycins 1			
Tetromycins 3			
-	*Chaetomium* sp.	*T.b. rhodesiense*	Xanthone (heterocyclic-substituted analogues)
Tryptophol	*Ircinia spinulosa*	*T.b. rhodesiense*	Alkaloids (indole-)
3-formylindole	*Anthiphates* sp	*T. cruzi*	Alkaloids (indole-)
3-hydroxyacetylindole			
*N*-acetyl-β-oxotryptamine			
Opacalines A	*Pseudodistoma opacum*	*T.b. rhodesiense*	Alkaloids (alkylguanidine-substituted β-carboline-)
Opacalines B			
Opacalines C			
Amino-1-(aminoimidazoyl)-prop-1-ene	*Agelas oroides*	*T.b. rhodesiense* and *T. cruzi*	Bromopyrrole derivatives
Oroidin trifluoroacetate salt	*Agelas oroides*	*T.b. rhodesiense*	Bromopyrrole derivatives
Longamide B	*Agelas dispar*	*T.b. rhodesiense*	Alkaloids
Longamide A	*Agelas longissima*	*T.b. rhodesiense*	Alkaloids
Oroidin dimer dibromopalau’amine	*Axinella verrucosa*	*T.b. rhodesiense*	Alkaloids
Sceptrin	*Agelas sceptrum*	*T.b. rhodesiense*	Alkaloids
Agelasine D	*Agelas nakamurai*	*T.b. brucei* and *T. cruzi*	Terpenes (bicyclic diterpenoid purine)
Convolutamines I	*Amathia tortusa*	*T.b. brucei*	Alkaloids (brominated β-phenyl ethylamine-based)
Convolutamines J			
Iotrochotamide A	*Iotrochota* sp.	*T.b. brucei*	Amino acids (cinnamoyl amino acids)
Iotrochotamide B			
Lepadins D	*Didemnum* sp.	*T.b. rhodesiense* and *T. cruzi*	Alkaloids (decahydroquinoline)
Lepadins E			
Lepadins F			
Viscosamine	*Haliclona viscosa*	*T.b. brucei*	Alkaloids (3-alkylpyridinium)
Fascaplysin	*Hyrtios* cf. *erecta*	*T.b. rhodesiense*	Alkaloids (pentacyclic bis-indole)
Ascididemnin	*Polysyncraton echinatum*	*T.b. brucei*	Alkaloids (pyridoacridines)
12-deoxyascididemnin			
-	*Aspergillus fumigatus*	*T.b. brucei*	Alkaloids (dimethylthio, spiro-pentacyclic and fused penta- and hexacyclic diketopiperazines)
Chaetocin	*Nectria inventa*	*T. cruzi*	Alkaloids (dimethylthio and two disulfide diketopiperazines)
Verticilin B			
Venturamides A	*Oscillatoria* sp	*T. cruzi*	Cyclic peptides
Venturamides B			
Aerucyclamides B	*Microrcystis aeruginosa*	*T.b. rhodesiense*	Alkaloids
Aerucyclamides C			
Almiramides B	*Lyngbya majuscula*	*T.b. brucei*	Alkaloids
Almiramides C			
-	*Liriodendron tulipifera* and *Lychnophora diamantinana* and *Viguiera robusta* and *Eremanthus goyazensis* and *Helianthus tuberosus*	*T.b. rhodesiense*	Sesquiterpenes (lactones)
8-epixanthatin 1,5-epoxide		*T.b. rhodesiense*	Sesquiterpenes (lactones)
Sinefungin	*Streptomyces grizeolus* and *S. incarnates*	*T. brucei* and *T. congolense* and *T. vivax*	-
Aculeatin D	*Amomum aculeatum*	*T. brucei rhodesiense* trypomastigotes	Aculeatins
Ferruginol	*Craniolaria annua*	*T. cruzi* trypomastigotes and epimastigotes	Abietanes (phenolic)
Montbretol			
α-solamargine	*Solanum lycocarpum* and *Solanum palinacanthum*	*T. cruzi*	Alkaloids (glycoalkaloids)
α-solasonine			
